# Two cases of type 2 diabetes mellitus successfully treated with probiotics

**DOI:** 10.1002/ccr3.3354

**Published:** 2020-09-22

**Authors:** Néstor Cardinali, Carlos Bauman, Facundo Rodriguez Ayala, Roberto Grau

**Affiliations:** ^1^ Instituto Cardinali de Nutrición Rosario Argentina; ^2^ Facultad de Ciencias Bioquímicas y Farmacéuticas Consejo Nacional de Investigaciones Científicas y Técnicas (CONICET) Universidad Nacional de Rosario Rosario Argentina; ^3^ Departamento de Micro y Nanotecnología Instituto de Nanociencia y Nanotecnología ‐ Comisión Nacional de Energía Atómica y CONICET Buenos Aires Argentina

**Keywords:** *Bacillus subtilis*, insulin resistance, probiotics, type 2 diabetes mellitus

## Abstract

The gut microbiota, and particularly probiotic bacteria, has emerged as a promising and novel intervention to fight the looming worldwide diabetes epidemic when combined with the appropriate medication. Herein, we report two cases of patient with type 2 diabetes refractory to conventional therapy that showed notable improvement after probiotic intervention.

## Graphical abstract

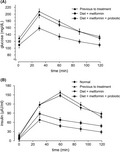

## 1 INTRODUCTION

Diabetes mellitus is a metabolic disease characterized by the presence of high blood glucose (hyperglycemia) either because insulin production is insufficient due to autoimmune destruction of insulin‐secreting β cells or pancreatitis—type 1 diabetes—and/or the body's cells do not respond properly to insulin (ie, insulin resistance), type 2 diabetes.^1^ The disease makes an important contribution to morbidity, loss of well‐being, and mortality worldwide. Currently, approximately 350 million people have diabetes mellitus worldwide, and this number is believed to reach almost 500 million people by 2030 (IDF, Diabetes Atlas). Among the two forms of diabetes, type 2 diabetes mellitus is the most common form of the disease and accounts for more than 90% of total diabetes worldwide. Worsening the global situation, there is a growing body of evidence linking type 2 diabetes mellitus with cardiovascular disease and dementia (eg, Alzheimer's disease).^2^ A growing number of type 2 diabetic patients do not respond properly to the available anti‐diabetic medication (eg, metformin, a common oral hypoglycemic agent).^1^ Therefore, there is an urgent need for the exploration and development of novel drugs and strategies against the onset and progression of diabetes mellitus.

The mechanisms and factors that influence the onset and progression of type 2 diabetes are subjected to intense research from many decades ago. However, the involvement of other actors, formerly neglected (eg, the gut microbiota), has emerged as alternative or cofounding strategies against the diabetic disease.^3^, ^4^ The gut microbiota (the trillions of microorganisms, mainly bacteria, residing in the host gut) influence the efficiency of energy extraction from ingested foods, time of food intestinal residence, mucosal immunity, intestinal permeability, and systemic inflammation, all factors involved in the triggering and progression of type 2 diabetes.^3^, ^4^ Interestingly, administration of probiotic bacteria has been reported as an approach to modulate the gut flora.^5^, ^6^ The Food and Agriculture Organization (FAO) and the World Health Organization (WHO) defined probiotics as live microorganisms, which when administered in adequate amounts and arriving alive to their sites of action (ie, the intestine), confer a health benefit on the host (FAO and WHO, 2002).^5^, ^6^ Here, we report two cases of type 2 diabetes mellitus, originally refractory to metformin treatment alone, which successfully responded to probiotics consumption.

## 2 CASE REPORTS

### 2.1 Case 1

**FIGURE 1:**
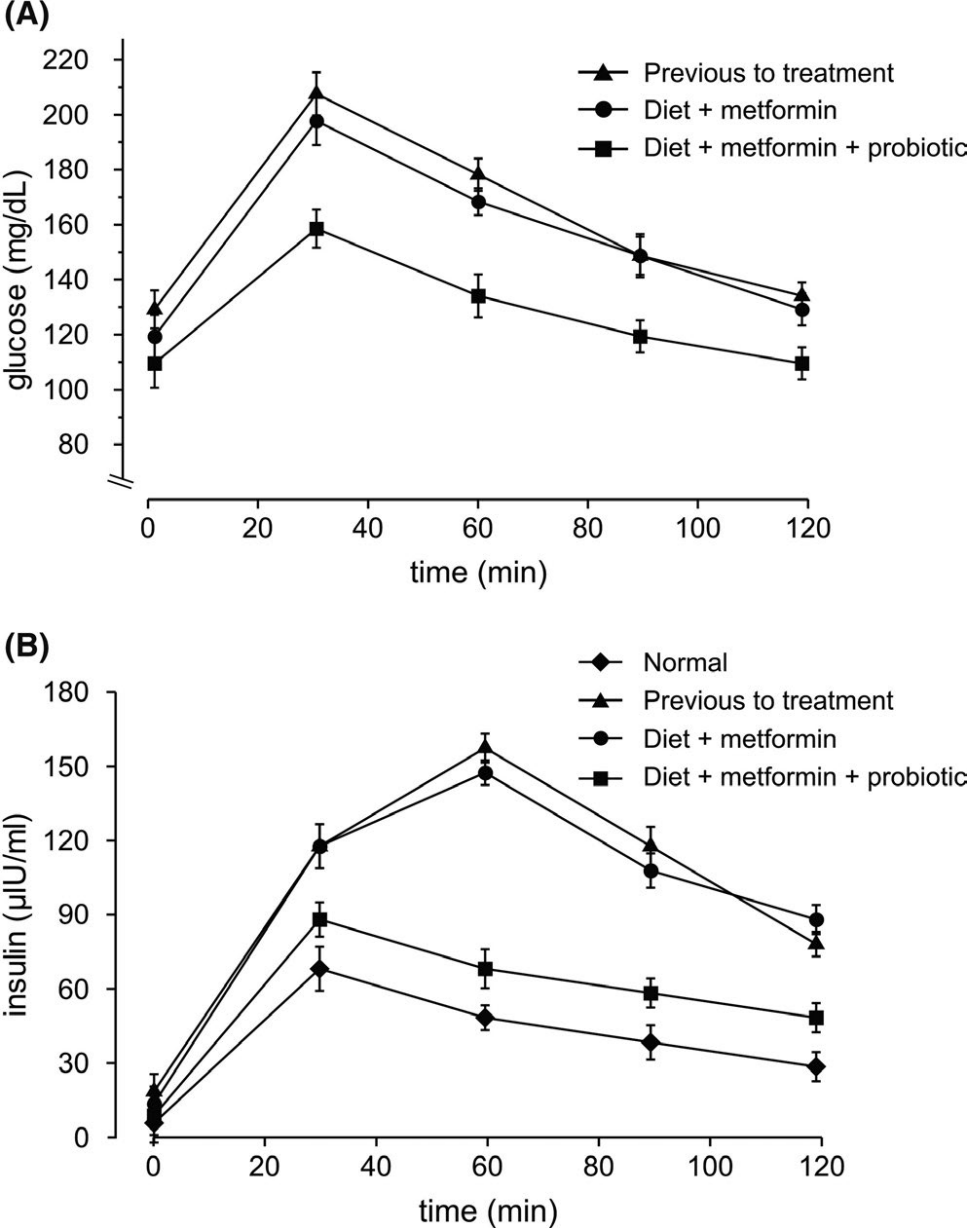
Probiotic *B subtilis* natto DG101 reduces glucose and insulin levels in a 71‐y‐old woman with type 2 diabetes mellitus combined with hypothyroidism, hypertension, and obesity (Case 1). Serum concentrations of glucose (A) and insulin (B) after an oral challenge with 75 g of glucose previous to patient treatment (triangles), after treatment with diet plus 850 mg/day metformin during 8 mo (circles), and after treatment with diet plus 850 mg/day metformin plus daily probiotic consumption during 4 mo (squares) are shown. In A, the expected glucose levels in a healthy patient should be lower than 140 mg/dL. Also shown is the expected variation of insulin concentrations in a healthy patient (B, diamonds). Values are presented as the mean ± SEM

A 71‐year‐old woman diagnosed with type 2 diabetes mellitus with a fasting blood glucose level of 118 mg/dL, (normal value ˂100 mg/dL) and insulin 14.4 µIU/mL (normal value ˂7 µIU/mL) was admitted in our institution for professional assistance in December 2018. Her body weight was 90.3 kg (body mass index, BMI: 32 kg/m^2^) and body fat 42.7%. The patient also suffered of hypothyroidism and hypertension, which were medicated and controlled. To corroborate the diabetic state of the patient, we measured her sugar tolerance (glucose and insulin levels) after an oral challenge with 75 g of glucose and the levels of glycosylated hemoglobin (HbA1c). The obtained results showed that effectively, the patient was hyperglycemic (Figure 1A, triangles) and hyperinsulinemic (Figure 1B, triangles). The insulin resistance index of the patient was calculated by using the homeostasis model assessment (In HOMA‐IR) and gave a value of 8.2 (normal values ˂2.3). According to the diabetic state, the obtained value of HbA1c was 6.3% (normal values ˂5.7%) (Table 1, Case 1). The prescribed treatment consisted in 850 mg/day of metformin and dietary intervention with a low‐fat/low‐caloric diet based on proteins and carbohydrates of low glycemic index. At the time of this treatment, the patient had normal hepatic function, AST: 25 (10‐40) IU/mL, and renal function, creatinine: 0.71 (0.45‐0.82) mg/dL. Diabetic complications (eg, retinopathy, dermopathy or neuropathy) were not observed. The medication for the preexisting hypothyroidism and hypertension was maintained throughout the intervention. The treatment was followed during 8 (eight) months with by‐monthly controls. After this period (August 2019), the patient did not show significant improvement in her diabetic situation, despite she was committed to and attained whit the treatment (Figure 1A‐B, circles). The blood levels of glucose and insulin remained high and the HOMA‐IR value was 7.9. Accordingly, the high levels of HbA1c remained unmodified (Table 1, Case 1). At this time, the body weight and body fat of the patient were 91.5 kg and 43.1%, respectively. Because of the treatment failure, we decided to incorporate the daily consumption of probiotic as a nutritional complement to the anti‐diabetic therapy. For our study, we selected the probiotic spore‐forming bacterium *Bacillus subtilis* natto DG101.^7^ This probiotic bacterium is originated from the millennial Japanese natto food that is worldwide reported for producing different healthy effects on consumers,^8^ and we were intrigued to know if the natto strain might control the blood sugar and insulin levels. Therefore, the patient was prescribed with a daily dose of 2 mL (approximately 40 drops) of *B subtilis* natto DG101 at a concentration of 1 × 10^8^ CFU (Colony Forming Units) per mL and 850 mg/day of metformin plus the original low‐fat/low‐caloric diet based on proteins and carbohydrates of low glycemic index. After 4 (four) months of treatment with metformin, modified diet and probiotic supplement, the blood levels of glucose and insulin decreased significantly to near normal values (95 mg/dL and 6.5 µIU/mL, respectively; Figure 1A‐B, squares) and the HOMA‐IR index decreased to 3.32. Accordingly, the levels of HbA1c decreased until reach a nearly normal value at the end of treatment (Table 1, Case 1). The body weight and body fat of the patient at the end of treatment were 89.5 kg and 41.1%, respectively. During the period of treatment, the patient had normal liver and renal functions, and no adverse events were observed.

**TABLE 1:** Probiotic *B subtilis* natto DG101 reduces HbA1c levels in a 71‐y‐old woman with type 2 diabetes mellitus combined with hypothyroidism, hypertension, and obesity (Case 1) and in a 47‐y‐old woman with type 2 diabetes mellitus combined with obesity (Case 2)

Condition	HbA1c levels (%)	
	Case 1	Case 2
No treatment	6.3 ± 0.35	7.4 ± 0.30
After treatment 1	6.3 ± 0.37	7.4 ± 0.32
After treatment 2	5.1 ± 0.31	5.6 ± 0.33

Note

Serum concentrations of HbA1c are shown as percentage in three conditions: without treatment, after treatment 1 (modified diet plus metformin), which lasted 8 mo and 12 mo for Cases 1 and 2, respectively; and after treatment 2 (modified diet plus metformin plus daily probiotic consumption), which lasted 4 mo for Cases 1 and 2. Percentage values are presented as the mean ± SEM.

### 2.2 Case 2

**FIGURE 2:**
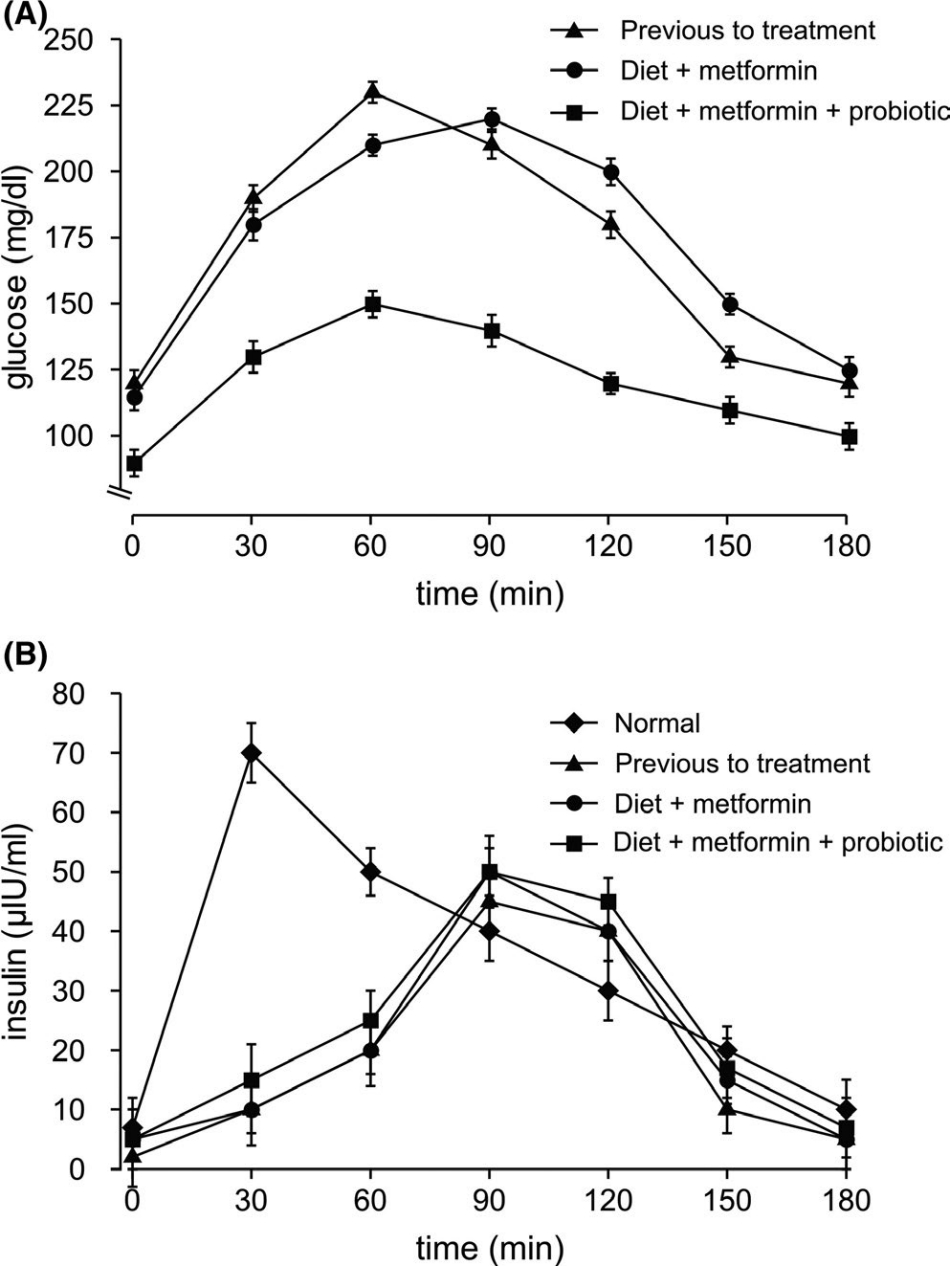
Probiotic *B subtilis* natto DG101 reduces blood glucose levels without insulin level increase in a 47‐y‐old woman with type 2 diabetes mellitus combined with obesity (Case 2). Serum concentrations of glucose (A) and insulin (B) after an oral challenge with 75 g of glucose previous to treatment (triangles), after treatment with diet plus 1 g/day metformin during 1 y (circles), and after treatment with diet plus 1 g/day metformin plus daily probiotic consumption during 4 mo (squares) are shown. In A, the expected glucose levels in a healthy patient should be lower than 140 mg/dL. Also shown is the expected variation of insulin concentrations in a healthy patient (B, diamonds). Values are presented as the mean ± SEM

A 47‐year‐old woman with a body weight of 98.5 kg (BMI: 39 kg/m^2^) and body fat 48.5% and, no liver or renal function complications (AST and creatinine values of 30 IU/mL and 0.75 mg/dL, respectively), was first admitted in our institution in April 2017. Her glucose and insulin blood basal levels were 240 mg/dL and 5.0 µIU/mL, respectively. The temporal glucose and insulin values after an oral challenge with 75 g of glucose are showed in Figure 2A‐B (triangles) and the HbA1c value was 7.4% (Table 1, Case 2). These results confirmed the type 2 diabetes state with hyperglycemia (Figure 2A, triangles), and hypoinsulinemia (reduced insulin response during oral glucose‐tolerance test, Figure 2B, triangles). The patient was medicated with 1 g/day of metformin and a low‐fat/low‐caloric diet based on proteins and carbohydrates of low glycemic index. The treatment was followed during 1 (one) year with by‐monthly controls. After the year, the patient increased her body weight and body fat (99.1 kg and 50.5%, respectively). Importantly, the diabetic state of the patient did not improve (Figure 2A‐B, circles) and glucose and insulin levels remained pathologic (220 mg/dL and 4.9 µIU/mL, respectively). In agreement with these results, the levels of HbA1c after the 1 year treatment remained abnormally high (Table 1, Case 2). Therefore, we prescribed a daily dose of 2 mL (approximately 40 drops) of *B subtilis* natto DG101 at a concentration of 1 × 10^8^ CFU (Colony Forming Units) per mL, 1 g/day of metformin and the same type of low‐fat/low‐caloric diet based on proteins and carbohydrates of low glycemic index, plus bimonthly controls. Interestingly, after 4 months of treatment with the probiotic supplement (plus metformin and the modified diet), the blood levels of glucose decreased to normal values, 95 mg/dL without an increase in insulin production, (Figure 2A‐B, squares), and the HbA1c level decreased to a normal value (Table 1, Case 2). The body weight and body fat of the patient at the end of treatment were 98.0 kg and 47.2%, respectively. During the period of treatment, the patient had normal liver and renal functions, and no adverse events were observed.

## 3 DISCUSSION

There is abundant scientific evidence originated from extensive in vitro cell line and animal studies suggesting that beneficial members of the gut flora and probiotics do have a real potential to prevent and reduce the severity of type 2 diabetes mellitus and other metabolic diseases.^3^, ^4^, ^5^, ^6^ However, the human clinical trials carried out during the last years could not conclusively support the health claim about probiotic efficacy in diabetic therapy.^5^, ^6^ One important weaknesses that we observe in these clinical studies are not taking into consideration the viability of the probiotics (the actual number or amount of alive probiotic) at the time of consumption. This is because probiotic efficiency depends on the ingestion of a proper amount (above a minimum number) of living probiotic bacteria. Effectively, the main probiotics used in former clinical trials for the treatment of diabetes belong to the genera of *Lactobacillus* and *Bifidobacterium* (collectively named as lactic acid bacteria, LAB).^9^, ^10^, ^11^, ^12^ All the members of the LAB group are extremely sensitive to atmospheric oxygen because they are anaerobic.^8^ The lethal effect of oxygen on LAB could have decreased the number of active (live) probiotics, from the time elapsed from its formulation to the moment of its consumption during the clinical trial, to a number or amount below the threshold required for the probiotic effectiveness. Besides LAB, there is another type of probiotics not affected by oxygen levels or other stressors. They belong to the *Bacillus* genus (ie, *B subtilis*, *B clausii*, and *B coagulans*).^13^ These bacilli have the property to form resistant spores that are 100% viable (alive) at the time of consumption, and capable of successfully traversing the acidic environment of the stomach, reaching the intestine, and germinating there to give rise to the active form of the probiotic and to an effective concentration.^13^ Here, we used the probiotic bacterium *B subtilis* natto DG101^7^, ^8^ as a possible co‐adjuvant therapy to treat diabetic people that do not respond properly to medication. Also important for the probiotic effectiveness, *B subtilis* is able to form robust and long‐lasting biofilms (ie, biopellicle) in the gut environment, a physiological property (absent in most LAB) that has been reported as crucial for the production of the beneficial effects of probiotic *B subtilis* on eukaryotic host.^14^, ^15^, ^16^, ^17^

Here, we presented two cases of type 2 diabetes mellitus in obese patients with poor control of the disease. Obesity is a strong risk factor for diabetes onset and progression. The patient 1, an obese 74‐years‐old woman, with hyperglycemia and hyperinsulinemia, produced a large amount of insulin but the increased blood concentration of insulin was not sufficient to meet the increased demands impose by obesity and insulin resistance. This patient did not respond to the combined treatment of metformin and a low caloric diet, but her glucose levels (and HbA1c values) successfully recovered nearly to the normal ones after the incorporation of the probiotic *B subtilis* DG101 to the treatment (Figure 1A‐B, squares, and Table 1, Case 1). The insulin production after an oral challenge with glucose shifted from a pathological insulin upregulation to a kinetic of insulin liberation very close to the one expected from a normal person (Figure 1B, squares). Interestingly, despite the lower levels of secreted insulin, the blood glucose values decreased to almost normal levels (Figure 1A, squares) and the HbA1c value was normal (Table 1, Case 1). One scenario for the outcome result is that once the probiotic spores germinate in human gut and form a biofilm, a signal molecule produced by them, reaches blood stream and stimulates glucose consumption and/or glucose transportation inside the cells (decreasing the blood glucose levels), an effect that would produce (as it is observed in Figure 1B, squares) a lower liberation of insulin from insulin‐synthetizing β cells in the pancreas. One small signaling molecule produced by *B subtilis* is the quorum‐sensing (QS) pentapeptide CSF (Competence Sporulation Factor).^18^ CSF is internalized via the mammalian oligopeptide transporter OCTN2 expressed in intestinal cells, where it induces the production of low molecular weight chaperones required to maintain protein homeostasis and the p38 MAPK and AKT survival pathways.^19^, ^20^ We hypothesize that CSF (or other QS molecule produce by *B subtilis*)^21^ might participate in the anti‐diabetic effect of the probiotic, a hypothesis that needs to be further investigated.

In the second case, an obese patient with hyperglycemia and pancreatic compromise (decreased insulin production by pancreatic β cells), the probiotic incorporation to the treatment decreased the blood glucose values to almost normal levels (Figure 2A, squares) without affect the kinetic of poor insulin production of the patient (Figure 2B, squares). According to these results, the HbA1c levels decreased from an initial value of 7.3% to a normal value of 5.6% after 4 (four) months of the combined (metformin plus diet plus probiotic) treatment (Table 1, Case 2). These results also support the hypothesis that the anti‐diabetic effect of the probiotic was not due to its action at pancreatic level (eg, regulating insulin production) but on the cellular uptake of glucose (see above).

There is a growing amount of evidence supporting that the subdivision of diabetes into type 1 and type 2 forms represents the extremes of a wide range of diabetic disorders, making this pathology much more heterogeneous and complex than the simplified picture of type 1 and type 2 diabetes.^1^, ^2^ In one of the intermediate forms of diabetes, there is a defective control of insulin secretion, wherein β cells produce insulin, but they respond poorly to increases in plasma glucose concentrations (Case 2). The presented results open the possibility that the spore‐forming probiotic *B subtilis* might work, directly or indirectly, influencing the cellular glucose transporters and transforming them into more sensitive or better responders to the blood circulating levels of insulin. However, since the complexity of the diabetes disease, the exact mechanism(s) of probiotic action against type 2 diabetes mellitus have to be worked out and required intense future research.^1^, ^15^ At the moment, the present results argue for the beneficial effect that probiotic *B subtilis* possesses for the treatment of type 2 diabetes mellitus when combined with appropriate medication.

## CONFLICT OF INTEREST

The authors declare that they have no competing interests.

## AUTHOR CONTRIBUTION

RG: reviewed, revised, and supervised the drafting of the manuscript. NC: is the primary nutritionist, treated the patients, and drafted the manuscript with RG. FRA and CB: were responsible for performing a literature search, drafting, and revising the manuscript. All authors: read and approved the final manuscript.

## ETHICAL APPROVAL

Agencia Santafesina de Seguridad Alimentaria (ASSAL) approved probiotic *Bacillus subtilis* DG101 use for human beings (RNPA: 21‐119482).
